# A simple method to distinguish light scattering from light absorption by nanoparticles†

**DOI:** 10.1039/d5na00194c

**Published:** 2025-09-24

**Authors:** Arianna Menichetti, Dario Mordini, Enrico Rampazzo, Agata Pane, Silvia Vicenzi, Vasilis Petropoulos, Giulio Cerullo, Fabrizio Mancin, Marco Montalti

**Affiliations:** a Dipartimento di Chimica “Giacomo Ciamician” Via Selmi 2 40126 Bologna Italy marco.montalti2@unibo.it; b Dipartimento di Fisica, Politecnico di Milano Milano 20133 Italy; c Department of Chemical Sciences, University of Padova Via Marzolo 1 35131 Padova Italy fabrizio.mancin@unipd.it

## Abstract

Photothermal efficiencies of natural and biomimetic melanin nanoparticles are compared. In order to justify the different behaviour, a preliminary investigation of the light scattering and light absorption properties of the NPs was performed using a simple new method based on a conventional fluorometer without the use of any integrating sphere.

## Abstract 2

Biomimetic and natural melanin nanoparticles show different photothermal efficiencies. The difference is due to different light absorption/light scattering efficiencies.

## Graphical abstract

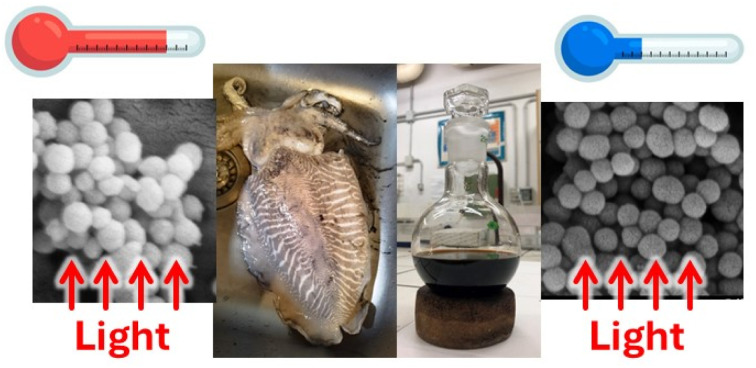

## Introduction

**Fig. 1:**
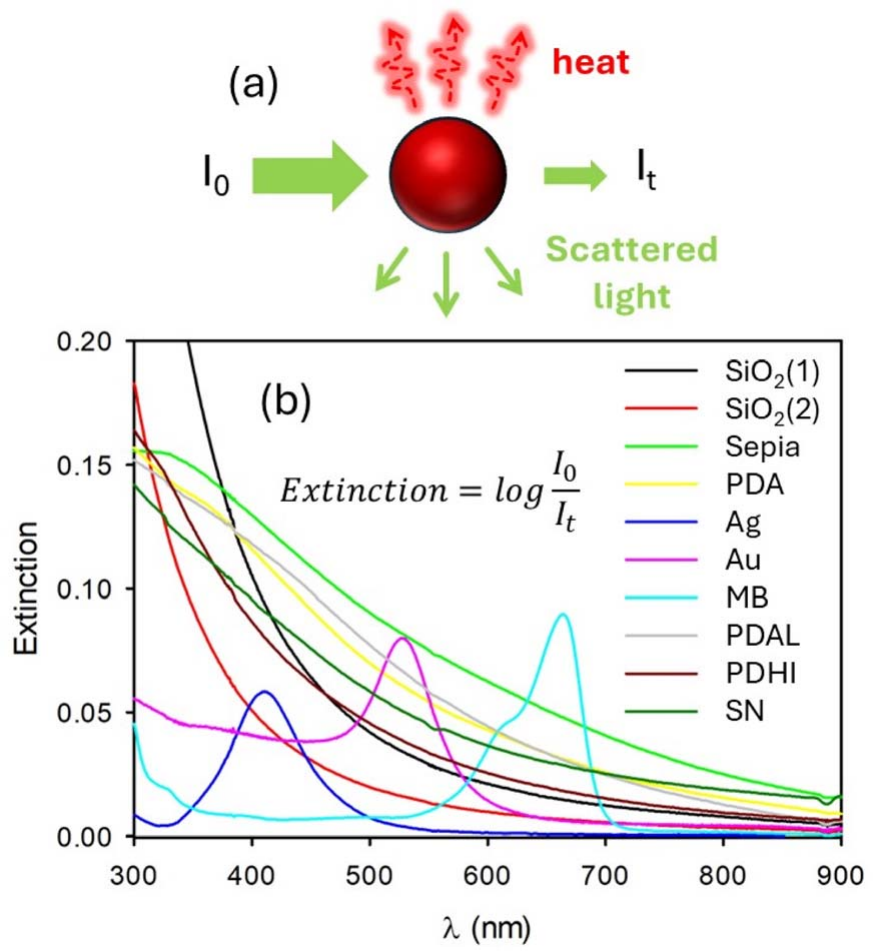
(a) Scheme of the light absorption and light scattering by NPs. (b) Extinction spectra of different kinds of NPs in a water suspension. SiO_2_ NPs were suspended at two different concentrations 1 and 2. Au corresponds to gold NPs and Ag to silver NPs. PDA represents polydopamine NPs while the Sepia sample contains melanin NPs obtained from the ink of cuttlefish (*Sepia officinalis*). MB is a water solution of methylene blue. PDAL is a suspension of PDA NPs with larger diameter with respect to PDA. PDHI resulted from oxidative polymerization of DHI NPs while SN is the supernatant obtained by the synthesis of PDA.

Nanoparticles (NPs) find applications in fields of high social and economic impact including medicine,^1–8^ energy conversion and storage,^9–12^ environmental remediation,^13,14^ and artwork preservation.^15^ Many of these applications are based on the interaction of NPs with light and in particular they exploit the photothermal effect, that is, the conversion of light energy into heat.^16,17^ For this use, absorption and scattering of light produce very different effects and nano-objects larger than molecules,^18^ hence NPs not only absorb light but also scatter light with good efficiency.^19–21^ As shown in Fig. 1a, both processes produce an overall light attenuation effect (extinction) but only in the case of light absorption is heat generation expected to occur. Besides this, while quantifying the extinction spectrum of a NP suspension by UV-Vis spectrophotometry is very simple, methods for discriminating the absorption from the scattering component generally are based on the use of a spectrofluorometer equipped with an integrating sphere.^22,23^ We would like to stress that typically, spectrofluorometers just collect light at 90° with respect to the excitation and do not have any integrating sphere.^24–26^ More in general, reported methods for discriminating between absorption and scattering by NPs require a specific set-up and experience.^27^ Here we report a simple and effective method, based on the use of a conventional set-up, for distinguishing the absorption and scattering components of NP suspensions and use it to justify the different photothermal efficiencies of natural (Sepia) and biomimetic (polydopamine, PDA) melanin NPs.^28–30^ In order to understand better the behaviour of PDA, a second sample of NPs with larger diameter (PDAL) and a sample of the PDA polymer obtained as supernatant (SN) during PDA NP synthesis were also analysed. Additionally, a sample of NPs obtained by polymerization of 5,6-dihydroxyindole (PDHI) was also considered. We also apply this method to other relevant examples of NPs, like gold NPs^31,32^ and silver NPs.^33–35^ Our method is based on the use of commercial silica NPs as a reference and it considers elastic scattering (same wavelength as the excitation) in the Rayleigh regime (small NPs, hence with diameter <200 nm in the visible range *λ* > 400 nm). We would like to stress that a complete theory of scattering by particles was developed by Mie in 1908.^36^

## Results and discussion

The different NP suspensions were prepared by diluting ultrapure water concentrated suspensions. The extinction spectra of the analysed samples are shown in Fig. 1.

All these spectra present a contribution due to light absorption and a contribution due to light scattering. Two suspensions of commercial SiO_2_ NPs at different concentrations, named SiO_2_(1) and SiO_2_(2) were used as reference for discriminating the two parts considering that for SiO_2_ NPs extinction is due only to light scattering in the examined spectral range. The extinction spectra of these two silica NP suspensions are also shown in Fig. 1. The different suspensions presented different colours, in particular the purple colour of the gold NP suspension (Au NP) was due to the plasmonic band at 537 nm while the yellow colour of the silver NP suspension (Ag NP) was caused by the plasmonic band at 408 nm. Biomimetic melanin NPs (polydopamine, PDA) and natural melanin NPs, obtained from the ink of cuttlefish (*Sepia officinalis*), presented a brown-black colour because of their typical broad-band extinction spectra.^37–39^

In order to confirm the effectiveness of our method, a molecular dye (methylene blue, MB) in water solution was also analysed. The MB water solution presented the typical blue colour due to the π–π* electronic transition at 668 nm.

**Fig. 2:**
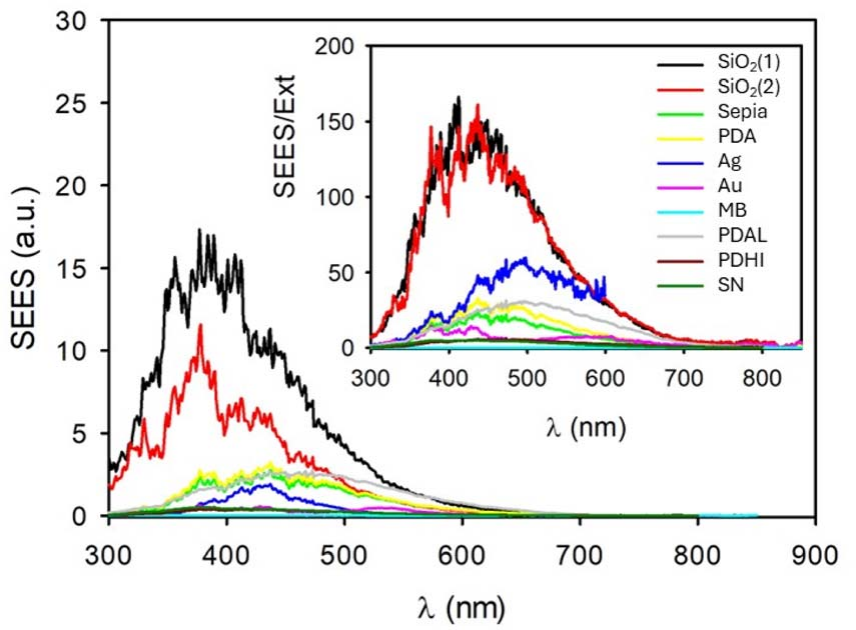
Synchronous excitation/emission spectra (SEES) in a water suspension. SiO_2_ NPs were suspended at two different concentrations 1 and 2. Au corresponds to gold NPs and Ag to silver NPs. PDA represents polydopamine NPs while the Sepia sample contains melanin NPs obtained from the ink of cuttlefish (*Sepia officinalis*). MB is a water solution of methylene blue. PDAL is a suspension of PDA NPs with larger diameter with respect to PDA. PDHI resulted from oxidative polymerization of DHI NPs while SN is the supernatant obtained by the synthesis of PDA. Inset: SEES divided by the corresponding extinction spectra. As discussed in the main text and in the ESI,† SEES/Ext is proportional to the scattering efficiency.

**Fig. 3:**
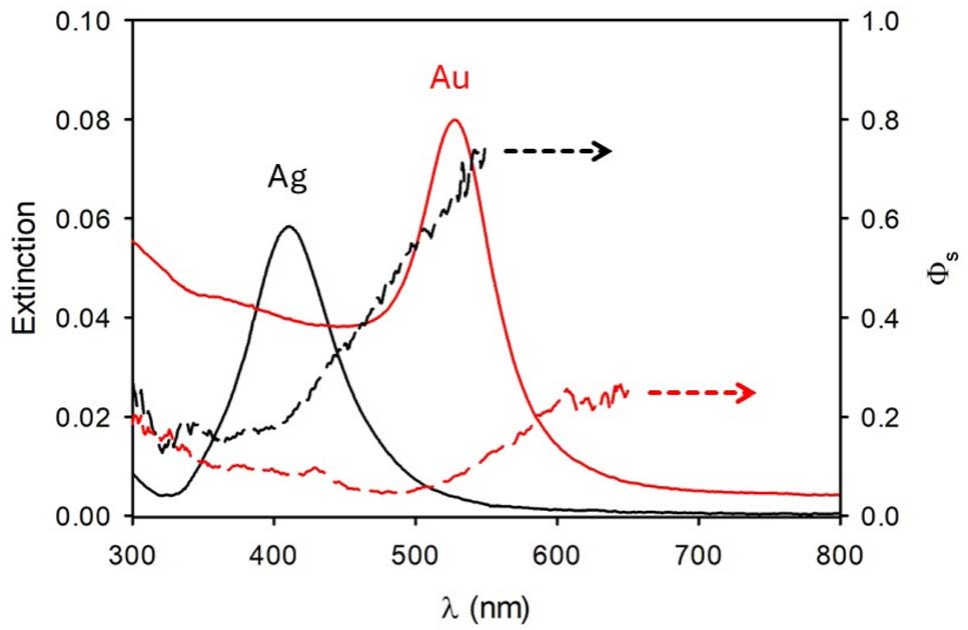
Extinction (continuous lines) and scattering (dashed lines) spectra of Ag NPs and Au NPs. The results show that extinction in the maximum of the plasmonic band (410 nm for silver and 528 nm for gold) is mostly due to light absorption.

**Fig. 4:**
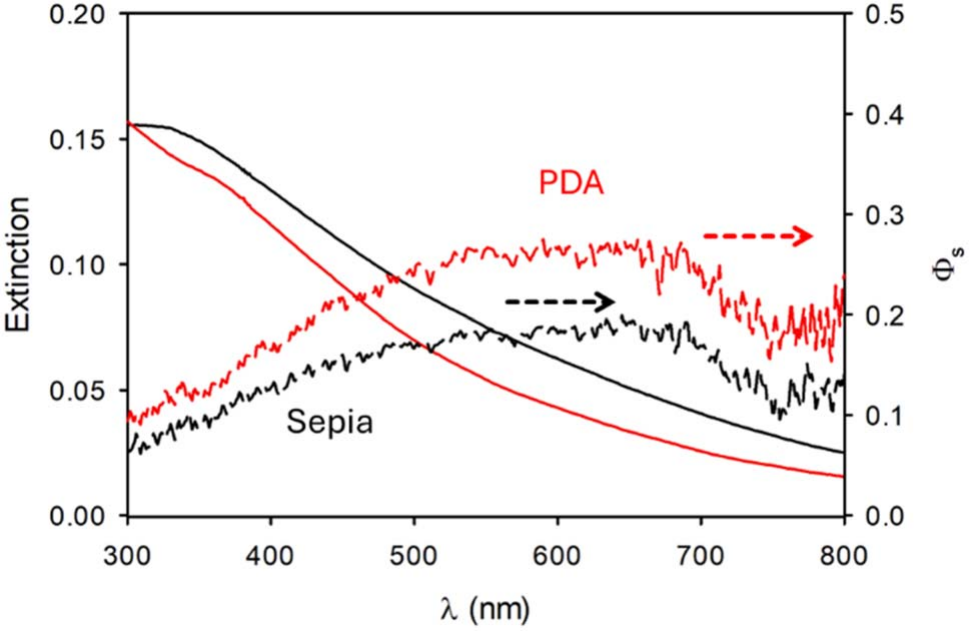
Extinction (continuous lines) and scattering (dashed lines) spectra of Sepia NPs and PDA NPs.

Light scattering spectra of the NP suspensions and of the MB solutions are shown in Fig. 2 and they were acquired on a conventional fluorometer using synchronous scanning. Briefly, in this acquisition mode both the excitation and emission monochromators move synchronously, hence they are positioned at the same wavelength. The scattering spectra shown in Fig. 2 are hence, more precisely, synchronous excitation/emission spectra (SEES). Since for low extinction value (<0.1) scattering intensity is expected to be proportional to the extinction, the SEES were divided by the corresponding extinction spectra and plotted in the inset of Fig. 2. These SEES/Ext spectra are expected to be a measurement of the intensity of the light scattered at different wavelengths but to be not dependent on the extinction value, hence they are a characteristic of the NP but concentration independent. The results confirmed this expectation; the inset of Fig. 2 in fact clearly shows that SEES/Ext spectra for the two SiO_2_ NP suspensions are very similar; besides, the concentration of NPs is different. This confirms that SEES/Ext is independent of the NP concentrations and just proportional to the scattering efficiency, *Φ*_s_ and to some experimental parameters which are the same for all the SEES of Fig. 2. The silica NP suspension can hence be used as a reference for calculating *Φ*_s_ of the other NP samples. SEES/Ext spectra can hence be used to calculate the scattering efficiency *Φ*_s_ in a very simple way according to the equation:
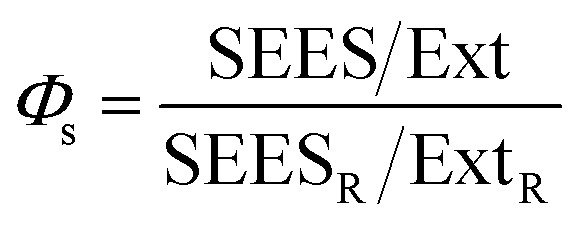
*Φ*_s_ values resulting from this simple calculation are shown in Fig. 3 for Ag and gold NPs while efficiency scattering values for Sepia NPs and PDA NPs are shown in Fig. 4.

Our results clearly confirm that extinction of Ag NPs and Au NPs at the wavelength corresponding to the maxima of the plasmonic bands (408 nm and 537 nm for silver and gold, respectively) is mostly due to absorption, the efficiency of scattering being as low as 20% and 9% for Ag and Au, respectively. In the case of the MB solution, scattering efficiency is close to zero, as expected in the case of a solution of small molecules which present extinction due only to light absorption.

Very interestingly, as shown in Fig. 4, in the case of Sepia and PDA NPs the contribution of the scattering to the extinction is significantly different though the composition of the two kinds of NP is quite similar.

Because of this important difference, different photothermal efficiencies are expected for the two kinds of NP. Non-radiative deactivation of the excited states, produced upon light absorption is, in fact, expected to produce an increase in the local temperature upon irradiation while light scattering is not.

In this framework the ability to discriminate between absorption and scattering is essential. For this reason, we compared the photothermal efficiency of sepia and PDA NPs.^40,41^

**Fig. 5:**
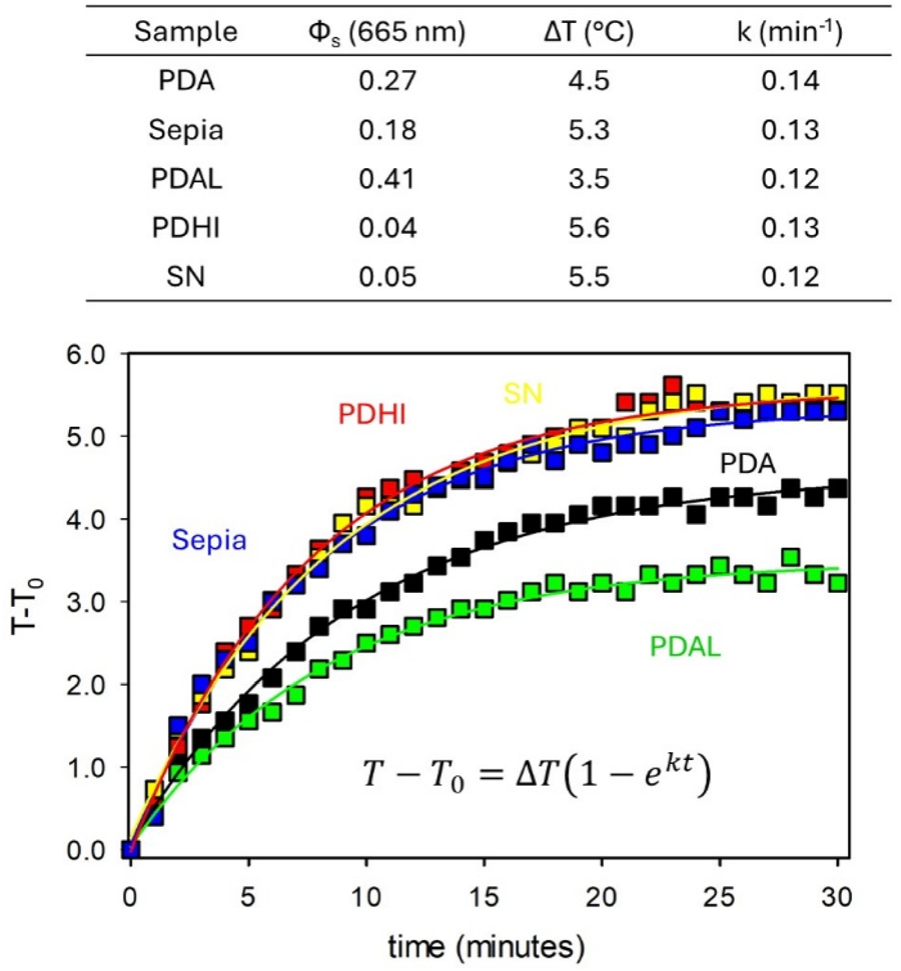
Temperature measured during irradiation at 665 nm of suspensions of PDA NPs, PDAL NPs, PDHI NPs, Sepia NPs and SN. All the suspensions showed the same extinction at 665 nm. Fittings of the experimental data according to the equation reported in the figure are plotted as continuous lines. Parameters resulting from the fitting are also shown in the table together with the light scattering efficiencies of the different samples at 665 nm.

The results are shown in Fig. 5. In particular, two NP suspensions having the same extinction value at 665 nm were irradiated, under the same conditions, with a focused 665 nm LED. The temperature of the suspensions was measured with a thermal camera and plotted as a function of the irradiation time as shown in Fig. 5. A similar experiment was performed, under the same conditions, for samples DHI NPs, SN and PDAL NPs. Experimental data were fitted according to the equation:^42^*T* − *T*_0_ = Δ*T*(1 − e^*kt*^)where *T*_0_ is the initial temperature, Δ*T* is the maximum temperature (*T*) increase and *k* is a kinetic parameter, discussed in the Experimental section (see the SI). The parameters obtained by the fitting were used to calculate the photothermal efficiency, which was 68% and 54% for Sepia NPs and PDA NPs, respectively.

More in general, as reported in the table of Fig. 5 a larger temperature increase was observed for the samples showing less scattering efficiency.

In particular, our results clearly demonstrate that Sepia NPs, which present a less efficient scattering of the light at the irradiation wavelength, absorb a higher amount of light energy than PDA NPs, though the extinction value is the same, and hence convert more light into heat exhibiting a more intense photothermal effect.

**Fig. 6:**
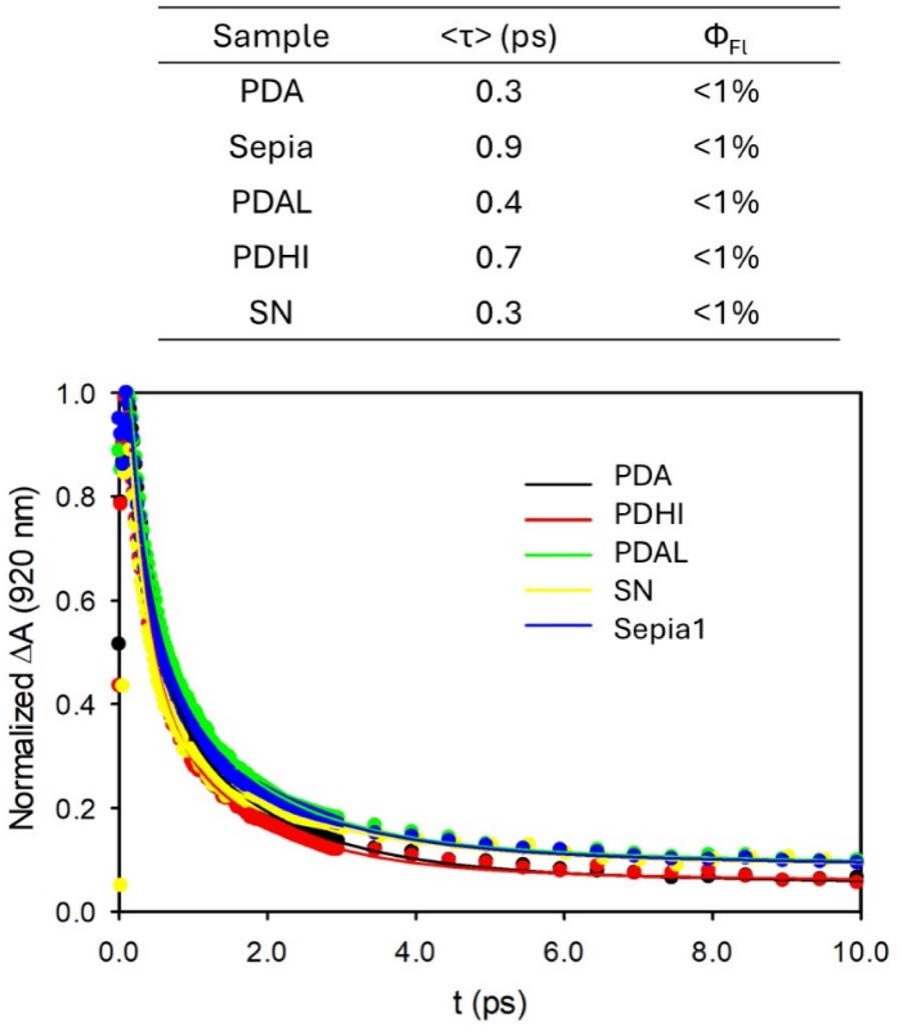
Ultrafast transient absorption traces upon excitation at 665 nm of PDA NPs, PDAL NPs, DHI NPs, Sepia NPs and SN. Fittings of the experimental data according to a tri-exponential decay model are plotted as continuous lines. Parameters resulting from the fitting are also shown in the table together with the fluorescence quantum yields.

In order to demonstrate that all the samples analysed from the photothermal point of view in Fig. 5 deactivate fast and almost completely *via* non-radiative processes, hence releasing heat and exhibiting a photothermal effect, we performed ultrafast transient absorption (UFTA) measurements and determined the fluorescence quantum yields as reported in Fig. 6.^43^

As summarized in the table of Fig. 6 all the samples deactivate fast, with an average lifetime of <1 ps and present a very low fluorescence quantum yield (<1%). Hence, for all the samples, absorbed energy is almost completely converted into heat rapidly. The differences in the photothermal efficiency are hence compatible with the different light scattering abilities.

## Conclusions

In conclusion, we present here a simple method for discriminating between light absorption and light scattering for NP suspensions. The method was applied to some important cases of inorganic and organic NPs and it allowed prediction of the different photothermal efficiency for different samples of natural and biomimetic melanin. This result is important for the design of new platforms for photothermal therapy and we believe that the application of our method to discriminate light absorption and light scattering can become an easy and widely accessible tool for the design and optimization of new devices based on NPs.

## Conflicts of interest

There are no conflicts to declare.

## Supplementary Material

NA-007-D5NA00194C-s001

## Data Availability

The data supporting this article have been included as part of the ESI.†
