# Supplementary material for: A simple method to distinguish light scattering from light absorption by nanoparticles
Source: Nanoscale Adv. 2025 Sep 24;7(21):6786–90. doi: 10.1039/d5na00194c (PMC12459033; doi:10.1039/d5na00194c)
Supplement: NA-007-D5NA00194C-s001 [file NA-007-D5NA00194C-s001.pdf]

Supplementary Information (SI) for Nanoscale Advances.
This journal is © The Royal Society of Chemistry 2025

A simple method to distinguish light scattering from light absorption by nanoparticles

Arianna Menichetti,[a] Dario Mordini,[a] Enrico Rampazzo,[a] Agata Pane, [a] Silvia Vicenzi,[a] Vasilis Petropoulos,[b] Giulio Cerullo,[b] Fabrizio Mancin,[c] Marco Montalti*[a]

[a] Dr. A., Menichetti, Mr. D. Mordini, Dr. Prof. E. Rampazzo, Ms. S. Vicenzi, Dr. Prof. M. Montalti
Department of Chemistry "Giacomo Ciamician"
University of Bologna
Via Selmi 2, 40126 Bologna, Italy
E-mail: marco.montalti2@unibo.it

[b] Dr. V. Petropoulos, Dr. Prof. G. Cerullo,
Dipartimento di Fisica, Politecnico di Milano, Milano 20133, Italy

[c] Dr. Prof. F. Mancin
Department of Chemical Sciences
University of Padova
Via Marzolo 1, 35131 Padova, Italy

Materials and methods

SiO_2 NP were purchased by Sigma Aldrich, Ludox TMA colloidal silica 34% water dispersion. Diameter d=20 nm (PDI=0.15)

Au NP were purchased by Sigma Aldrich (741981). Diameter d=56 nm (PDI=0.15).

Ag NP were prepared as previously reported.[1] Diameter d=67 nm (PDI=0.22)

Polydopamine (**PDA**) NP were prepared by the spontaneous oxidation of dopamine (DA) hydrochloride in aerobic and alkaline conditions. Initially, 40 mL of ethanol and 90 ml of deionized water were mixed in a 250 mL round bottom flask. 2 mL of 28-30% NH4OH were added to the flask and the mixture was stirred vigorously for 10 min. In the next step, a DA solution was prepared by solubilizing 400 mg of DA hydrochloride in 10 mL of deionized water. The DA solution was injected fast into the flask under vigorous stirring, final concentration of DA 14.9 mM. An immediate colour change was observed which evolved in time from light yellow to dark brown. The reaction was carried out for 24 hours under vigorous stirring. After 24 hours, the raw batch was centrifuged in order to separate the formed nanoparticles (NPs). In detail, the NPs were retrieved by centrifugation at 14000 rpm for 20 minutes. Then, they were washed with

deionized water three times at 10000 rpm and finally redispersed in the same solvent. After the centrifugation, the purified from the NPs PDA was kept.

SN is the supernatant resulting from the centrifugation of PDA and containing low molecular weight polydopamine.

Sepia melanin nanoparticles (NPs) were obtained by cuttlefish ink purification. Ink was extracted from the sack of cuttlefish and was diluted with MilliQ water. The obtained ink dispersion was placed in 10 ml Falcons and was centrifuged at 3.000 rpm for 10 minutes to remove residues (sand or parts of the sack of the fish). After the end of the centrifugation, the supernatant was kept and diluted with MilliQ water and centrifuged at 6500 rpm for 10 minutes. Then, the supernatant was removed and the pellets were further washed other 3 times with MilliQ water by centrifugation.

PDAL NP were prepared similarly to PDA but using a smaller amount of NH_4OH. Initially, 40 mL of ethanol and 90 ml of deionized water were mixed in a 250 mL round bottom flask 0.5 mL of 28-30% NH_4OH were added to the flask and the mixture was stirred vigorously for 10 min. In the next step, a DA solution was prepared by solubilizing 400 mg of DA hydrochloride in 10 mL of deionized water. The DA solution was injected fast into the flask under vigorous stirring, final concentration of DA 14.9 mM. The reaction was carried out for 24 hours under vigorous stirring. After 24 hours, the raw batch was centrifuged in order to separate the formed nanoparticles (NPs). In detail, the NPs were retrieved by centrifugation at 14000 rpm for 20 minutes. Then, they were washed with deionized water three times at 10000 rpm and finally redispersed in the same solvent.

PDHI NP were synthesized adapting the synthesis of PDA to the precursor 5,6-dihydroxyindole (DHI). Initially 2.1 mmol of DHI (313 mg) were solubilized in 40 mL of ethanol and 100 ml of deionized water were added in a 250 mL round bottom flask. 2 mL of 28-30% NH_4OH were then added to the flask and the mixture was stirred vigorously for 24 hours.

Dynamic light scattering (DLS)

Hydrodynamic diameter of the NP was determined by DLS. measurements were performed with Zetasizer Nano ZS Malvern using PMMA semi-micro cuvettes.

Measurements were repeated three times for each sample giving the results reported in table S1

Table S1. Triplicated DLS measurements

Sample	Diameter 1 (PDI)	Diameter 2 (PDI)	Diameter 3 (PDI)
PDA	175 nm (0.04)	179 nm (0.03)	181 nm (0.03)

Sepia	193 nm (0.04)	202 nm (0.04)	203 nm (0.04)
PDHI	Low signal	Low signal	Low signal
PDAL	297 nm (0.06)	305 nm (0.05)	301 nm (0.05)
SN	Low signal	Low signal	Low signal

We would like to stress that DLS measurement of weakly scattering samples like PDHI and SN are poorly significant.

UV-Vis extinction spectra (EXT)

The experiments were carried out in air-equilibrated solutions at 25 °C. UV–Vis extinction spectra were recorded using a Perkin-Elmer Lambda 40 spectrophotometer in the wavelength range of 300-900 nm, with PMMA cells having a path length of 1.0 cm

All the measurements were repeated three times and the average was shown in figure 1 in the main text and was used to calculate the scattering efficiency. In order to demonstrate the reproducibility of the results the three measurements performed for sample SiO_2(2) are shown in figure SI1

Synchronous excitation/emission spectra (SEES)

Synchronous excitation-emission spectra of the samples were determined using a FluoroMax-4 system (Horiba Scientific; Kyoto, Japan), in the wavelength range of 300-900 nm both for excitation and emission, using PMMA cells with a path length of 1.0 cm.

All the measurements were repeated three times and the average was shown in figure 2 in the main text and was used to calculate the scattering efficiency. In order to demonstrate the reproducibility of the results the three measurements performed for sample SiO_2(2) are shown in figure SI2

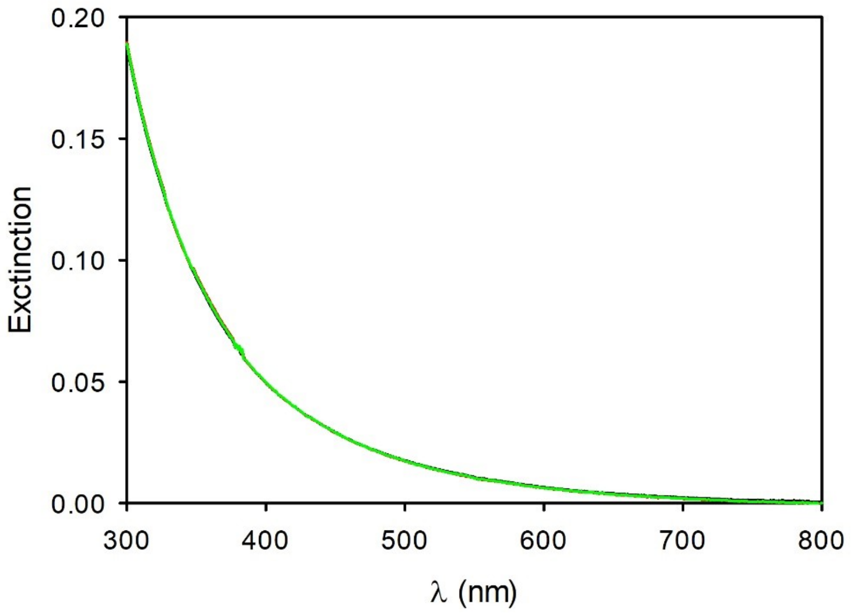

Figure SI1. Extinction spectra of a SiO_2 NP suspension. The spectrum was acquired 3 times. Measurements showed good reproducibility.

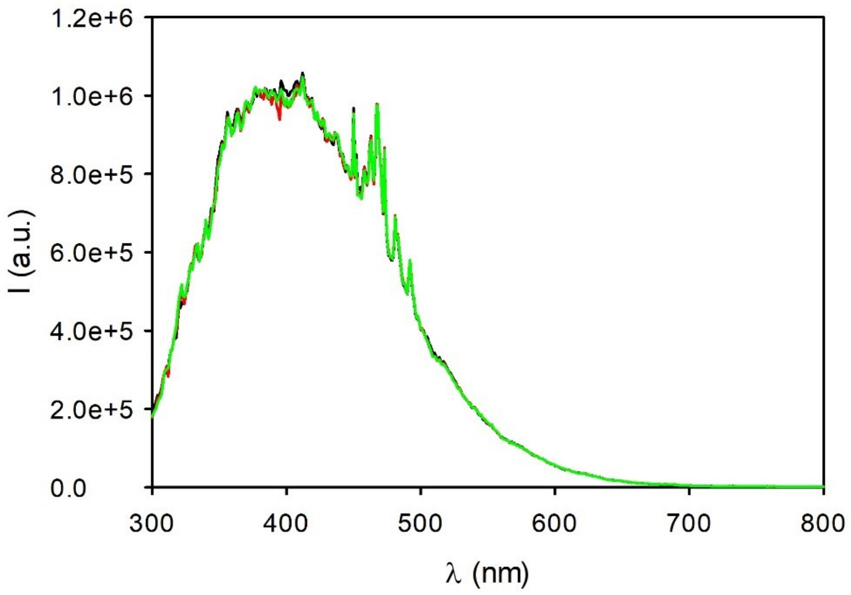

Figure SI2. SEES of a SiO_2 NP suspension. The spectrum was acquired 3 times. Measurements showed good reproducibility.

Ultra-fast transient absorption

Ultra-Fast Transient Absorption (UFTA) experiments were carried out using a laser system, which was based on an amplified Ti:Sapphire laser that generates 100-fs pulses at 800 nm with a repetition rate of 1 kHz. The beam was initially divided through a beam splitter. One part of the beam, also called pump beam, was used to pump a non-colinear optical parametric amplifier (NOPA) to generate ~100-fs laser pulses centered at 520 nm. Pump pulses were modulated by a mechanical chopper at 500 Hz repetition rate and were delayed in time with the use of a mechanical stage. The pump was focused on a 320 μm diameter spot under a fluence of 1.5 $\mu J/cm^2$ in order to minimize exciton-exciton annihilation processes. The other part of the beam was used to generate the probe pulses. The UFTA data were obtained with a magic angle (54.7˚) configuration between the pump and probe polarizations. Lastly, for the analysis a global analysis algorithm (Glotaran) was used.

Luminescence quantum yield determination

Luminescence quantum yields were measured against a standard of $[Ru(bpy)_3]^{2+}$ in aerated millipore water(Φ=0.04).[2] The emission intensity was corrected for inner filter effects. Experimental details for the photophysical measurements are reported elsewhere.[3]

Scattering quantum yield calculations

The intensity of light scattered at 90 degrees, measured in synchronous excitation/emission spectra (SEES) is proportional to the scattering quantum yield (Φ_S) and to the extinction (Ext) for low extinction value (Ext <0.1). Hence:

$SEES = K\Phi_S Ext$ (eq. 1)

Where K is a constant that depends on the experimental conditions that were kept unchanged in the different SEES acquisition.

For silica NP, used as reference (R) extinction is due only to the scattering and Φ_S=1 hence:

$$K = \frac{SEES_R}{Ext_R} \quad \text{(eq.2)}$$

For a generic sample, according to eq.1

$$\Phi_s = \frac{SES/Ext}{SES_R/Ext_R} \quad \text{(eq.3)}$$

Photothermal experiments

Irradiation was performed with an LED from OSRAM, LZ1-10R202-0000 at a controlled current of 1A. Light was focused with a lens, Thorlabs ACL25416U, Aspheric Condenser Lens, diameter 2.54 mm, f=16 mm, NA=0.79. Total power emitted by the focused beam was 0.43 Js^{-1}. Emission spectrum of the diode is shown in figure S1.

Temperature was measured using a thermos-camera Optris Xi 80.

Calculation of the photothermal conversion efficiency

The total energy balance of the system can be expressed as:

$$\sum_i m_i C_{Pi} \frac{dT}{dt} = Q_{NP} + Q_D - Q_s$$

.

Where m and C_p are respectively the mass and heat capacity of water, T the solution temperature, Q_{NP} is the energy inputted by NP, Q_D the baseline energy inputted by sample cell and finally Q_s is heat conducted away from the system surface by air.

The light-induced term, Q_{NP}, represents the heat dissipated by radiationless relaxation of PBI NPs excited states under the irradiation of 665nm LED:

$$Q_{NP} = I(1 - 10^{-A_{665}})\eta$$

Where I is the irradiation light power, η is the conversion efficiency from radiative energy to thermal energy, and A_{665} is PBI NP absorbance at wavelength 665 nm. In addition, Q_D source term expresses the heat dissipated from light absorbed by sample cell containing pure water.

Finally, Qs is given by the linear equation:

$$Q_S = hS(T - T_S)$$

Where h is the heat transfer coefficient, S is the surface area of sample cell and T_S is the ambient temperature of surroundings. For a defined irradiation power, the system heat input, ($Q_{NP} + Q_D$) resulted to be finite. Since the heat output (Q_S) increases linearly with temperature, the resulting system temperature will reach a maximum when the heat input and output are equal:

$$Q_{NP} + Q_D = Q_{s\,max} = hS(T_{max} - T_S)$$

Where $Q_{s\,max}$ is heat conducted away from the system surface by air at equilibrium temperature and T_{max} the equilibrium temperature. Heat conversion efficiency (η) can be determined rearranging former equations to get:

$$\eta = \frac{hS(T_{max} - T_S) - Q_D}{I(1 - 10^{-A_{460}})}$$

Where Q_D was measured to be ~ 0 Js^{-1}, ($T_{max} - T_S$) was 5.3 and 4.5 °C for Sepia and PDA respectively, $I(1 - 10^{-A_{660}})$ was 0.08 Js^{-1}. In order to get hS, a dimensionless driving force temperature θ is introduced:

$$\theta = \frac{T - T_S}{T_{max} - T_S}$$

And a sample system time constant τ_s expressed as:

$$\tau_s = \frac{\sum_i m_i C_{Pi}}{hS}$$

Which is substituted and rearranged in

$$\frac{d\theta}{dt} = \frac{1}{\tau_s}\left[\frac{Q_{NP} + Q_D}{hS(T_{max} - T_S)} - \theta\right]$$

At cooling stage of the solution, the LED was shut off, the $Q_{NP} + Q_D = 0$, reducing

$$\frac{d\theta}{dt} = -\frac{1}{\tau_s}\theta$$

And integrating, giving the expression:

$$\theta = e^{-\frac{t}{\tau_s}}$$

$$hS = \frac{\sum_i m_i C_{Pi}}{\tau_s}$$

Therefore, time constant for heat transfer from the system is determined to be τ_s = 420 s. In addition, m is 1.0 g and the C is 4.18 $Jg^{-1}K^{-1}$. Thus, the hS

$$hS = \frac{\sum_i m_i C_{Pi}}{\tau_s}$$

is deduced to be 0.020 W/°C and the photothermal conversion efficiency $\eta_{Sepia} = 68\%$ and $\eta_{PDA} = 54\%$.

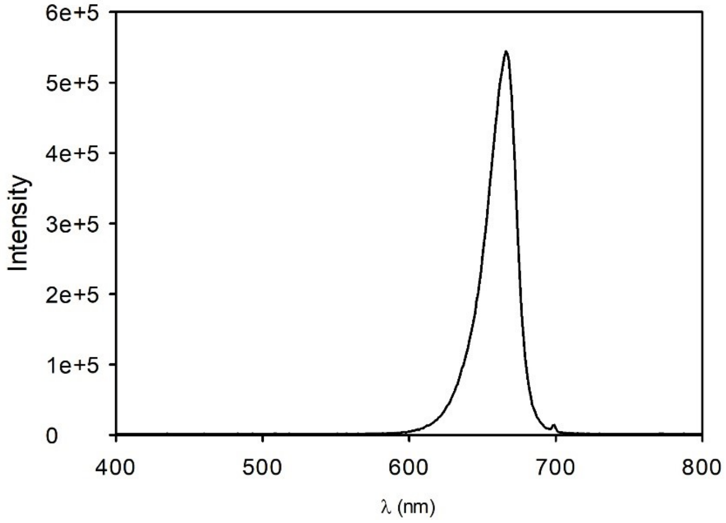

Figure SI3. Emission spectrum of the LED used for the photothermal experiments.

[1] H. Wang, X. Qiao, J. Chen, X. Wang, S. Ding, *Materials Chemistry and Physics* **2005**, *94*, 449-453.

[2] K. Suzuki, A. Kobayashi, S. Kaneko, K. Takehira, T. Yoshihara, H. Ishida, Y. Shiina, S. Oishi, S. Tobita, *Physical Chemistry Chemical Physics* **2009**, *11*, 9850-9860.

[3] M. Montalti, A. Credi, L. Prodi, M. T. Gandolfi, *Handbook of Photochemistry (3rd ed.)*, CRC Press, **2006**.
